# The isotopic signature of the “arthropod rain” in a temperate forest

**DOI:** 10.1038/s41598-021-03893-6

**Published:** 2022-01-10

**Authors:** Oksana L. Rozanova, Sergey M. Tsurikov, Marina G. Krivosheina, Andrei V. Tanasevitch, Dmitry N. Fedorenko, Vladislav D. Leonov, Alexander V. Timokhov, Alexei V. Tiunov, Eugenia E. Semenina

**Affiliations:** 1 A.N. Severtsov Institute of Ecology and Evolution, Russian Academy of Sciences, Leninsky Prospect 33, 119071 Moscow, Russia; 2 Department of Entomology, Faculty of Biology, Lomonosov Moscow State University, Leninskie Gory 1/12, 119234 Moscow, Russia

**Keywords:** Food webs, Ecological networks, Ecosystem ecology, Forest ecology, Stable isotope analysis

## Abstract

Forest canopy is densely populated by phyto-, sapro-, and microbiphages, as well as predators and parasitoids. Eventually, many of crown inhabitants fall down, forming so-called ‘arthropod rain’. Although arthropod rain can be an important food source for litter-dwelling predators and saprophages, its origin and composition remains unexplored. We measured stable isotope composition of the arthropod rain in a temperate mixed forest throughout the growing season. Invertebrates forming arthropod rain were on average depleted in ^13^C and ^15^N by 1.6‰ and 2.7‰, respectively, compared to the soil-dwelling animals. This difference can be used to detect the contribution of the arthropod rain to detrital food webs. Low average δ^13^C and δ^15^N values of the arthropod rain were primarily driven by the presence of wingless microhytophages, represented mainly by Collembola and Psocoptera, and macrophytophages, mainly aphids, caterpillars, and heteropterans. Winged arthropods were enriched in heavy isotopes relative to wingless specimens, being similar in the isotopic composition to soil-dwelling invertebrates. Moreover, there was no consistent difference in δ^13^C and δ^15^N values between saprophages and predators among winged insects, suggesting that winged insects in the arthropod rain represented a random assemblage of specimens originating in different biotopes, and are tightly linked to soil food webs.

## Introduction

Numerous feedback mechanisms link the above- and belowground components of ecosystems^1–3^. For instance, aboveground consumers of higher trophic levels receive energy subsidies from detrital food webs, which allows for a larger population of aboveground invertebrate generalist predators^4–6^. This phenomenon subsequently impacts plant productivity by controlling the populations of phytophages^7^. On the other hand, detrital food webs in soil generally based on dead plant tissues can be subsidized by other sources of nutrients and energy from above ground. In forest ecosystems, soil predators such as ants often forage in the canopy^8,9^, but the “arthropod rain,” i.e., various animals and their derivatives falling from the crowns^10^, may be a more important link connecting soil food webs and the canopy layer. However, only a few studies have thus far addressed the contribution of above-ground animals (likely belonging to grazing food webs) to belowground detrital food webs, although above-ground prey providing a subsidy to soil predators is likely to be a widespread phenomenon^11^.

Tree crowns are populated by phytophages and sapro- and microbiphages, as well as predators and parasitoids. Eventually, many crown inhabitants fall from the trees and become a food source for litter-dwelling predators, scavengers, and saprophages. Our first arthropod rain study in a temperate forest^12^ showed that the rain was taxonomically diverse: it comprised approximately 15 orders and more than 60 families of invertebrates. Some of these animals are slow-moving and defenseless and can be easily captured by soil predators. Moreover, approximately 28% of the total mass of arthropod rain is formed by dead animals and exuviae; this fraction can be consumed by saprophages such as collembolans. A few studies using stable isotope labeling of tree crowns confirmed these suggestions: invertebrate predators and saprophages inhabiting the upper layer of litter obtained the crowns’ isotopic label more often than the inhabitants of lower litter layers and soil^13,14^.

Theoretically, the contribution of the arthropod rain into the diet of soil-dwelling predators and saprophages can be estimated using the intrinsic difference in the isotopic composition of “green,” i.e., grazing, and “brown,” i.e., detrital, food chains^15^. It is well known that members of detrital food webs are 2–5‰ enriched in ^13^C relative to leaf litter^16,17^, while in the grazing food webs, the difference in δ^13^C values between green plants and phytophages typically does not exceed 1‰^18,19^.

Furthermore, many microarthropods inhabiting the canopy are likely microphytophages feeding on algae, lichens, and mosses^20^. Non-vascular plants and epiphytes, in general, typically have low δ^15^N values due to the assimilation of ^15^N-depleted compounds from wet atmospheric deposits^21–23^. Consequently, arthropods trophically linked to non-vascular plants are depleted in ^15^N relative to litter^24^. This depletion should result in the relatively low δ^15^N values in the arthropod rain. In contrast, most soil animals are strongly enriched in ^15^N compared to phytophages due to the accumulation of heavy nitrogen in microbial biomass at basal levels of detrital food webs^16^. Overall, due to the prevalence of macrophytophages and microphytophages in the arthropod rain, it can be expected to be depleted by 2–3‰ in ^13^C and ^15^N content relative to the animals belonging to detrital food webs in soil.

Although a considerable difference in resource base (and consequently in the isotopic composition) of the crown fauna and soil-dwelling species is expected, some members of the arthropod rain would, in fact, belong to detrital food webs. First of all, these are winged insects that could spend their early life stages or feed in the soil^25^. Second, besides winged insects, actively moving (climbing) specimens of wingless macrofauna (mostly predators like harvestmen, spiders, ants) move freely between the litter and the tree canopy connecting belowground and aboveground food webs^1,8^. Finally, detritus and small-scale detrital food webs can be quite abundant in the canopy, supporting a relatively rich fauna of typical microbivores and detritivores such as Oribatida or Collembola^26,27^.

Thus, the study of the isotopic composition of the arthropod rain would contribute to elucidating trophic relationships of its constituent invertebrates. Furthermore, it would allow us to assess the possibility of evaluating the contribution of arthropod rain to the nutrition of soil invertebrates.

In this study, we estimated the stable isotope composition of invertebrates forming arthropod rain in a temperate forest. Our main goal was to compare δ^15^N and δ^13^C values of the arthropod rain with those of soil- and litter-dwelling invertebrates. We hypothesized that (1) invertebrates forming arthropod rain are depleted in ^13^C and ^15^N compared to the soil-dwelling animals. We further proposed that (2) winged insects in the arthropod rain have on average higher δ^13^C and δ^15^N values than wingless invertebrates because the former are likely to have tight trophic connections with soil and detrital food webs.

## Results

The most numerous taxa in the arthropod rain were Collembola and Acari. Collembola were mainly represented by Entomobryidae, Sminthuridae, Dicyrtomidae, and Poduromorpha. Mites were mainly Trombidiformes, Gamasina, Astigmatina, and Oribatida. The most species-rich Insecta orders were Coleoptera and Diptera, forming the main part of the winged specimens. Numerous fly larvae were represented by 18 families, Mycetophilidae, Sciaridae, and Cecidomyiidae being most frequent. Coleoptera were represented by 29 families, and the most numerous were Staphylinidae, Ptiliidae, and Lathridiidae. Numerous aphids, some heteropterans, and relatively rare lepidopteran larvae were the most characteristic representatives of macrophytophages. Hymenoptera were represented mainly by parasitoid Mymaridae, Ceraphronidae, Diapriidae, and the rare wingless Formicidae. Spiders were represented by web-builders and striders; most species typically inhabit vegetation (Araneidae, Theridiidae, and Thomisidae including *Enoplognatha ovata* Clerck*, Xysticus* sp. and *Philodromus* sp.), but some litter-dwelling species (*Ceratinella brevis* Wider, Linyphiidae; *Ozyptila praticola* C. L. Koch, Thomisidae) were also present. Opiliones were not abundant, but accounted for a large proportion of the mass and were represented by *Mitopus morio* Fabricius and *Phalangium opilio* L. Data on the seasonal fluctuations in arthropod rain intensity are given in Rozanova et al.^12^.

Isotopic analysis of the arthropod rain revealed a large range of both δ^13^C and δ^15^N values. Mean isotopic composition of the main taxonomic groups and life stages of the arthropods, along with exuviae, frass, and various plant materials, are given in Table S1. The total ranges of litter-normalized Δ^13^C and Δ^15^N values of individual animals forming the arthropod rain (n = 730) were 13.7‰ (from − 3.4‰ in imago Anisopodidae, Diptera to 10.3‰ in imago Staphylinidae, Coleoptera) and 26.2‰ (from − 7.2‰ in Psocoptera to 18.7‰ in imago Figitidae, Hymenoptera), respectively. The Δ^13^C and Δ^15^N values of excrements (frass) and exuviae were within the range of arthropod δ values (Fig. S2).

**Figure 1:**
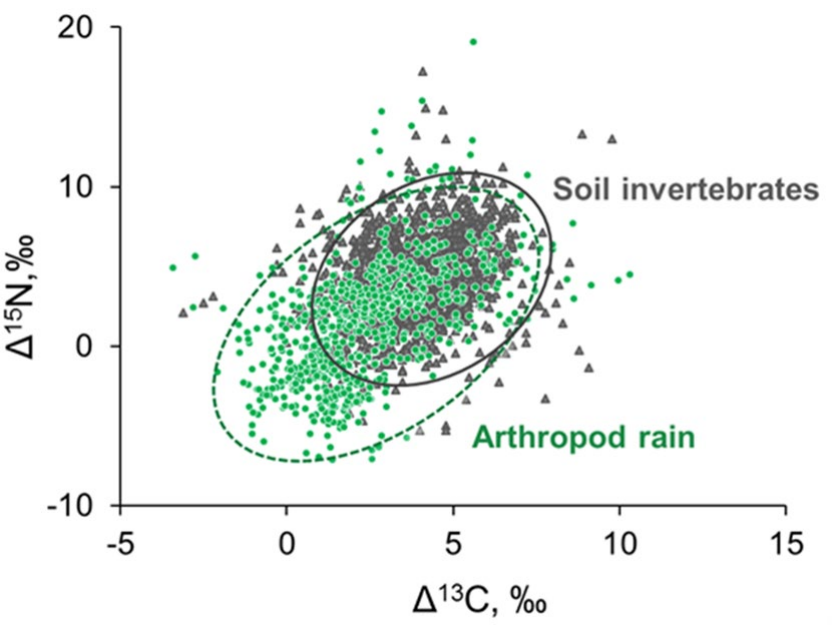
Litter-normalized isotopic composition (Δ^13^C and Δ^15^N values) of the arthropod rain (green dots and dashed line, original data, each point is one animal or several conspecifics, n = 730) and soil invertebrates (gray triangles and solid line, published data, points represent the mean value of species in different ecosystems, n = 1300). Standard ellipses show the 95% confidence interval.

The previously reported ranges of mean litter-normalized Δ^13^C and Δ^15^N values of soil invertebrates in temperate forests (n = 1300^16^) were similar to those of the arthropod rain. In the isotopic bi-plot (Fig. 1) nearly all soil invertebrates are found within the convex hull formed by the arthropod rain. Standard ellipses of the arthropod rain and soil invertebrates largely overlapped, but the arthropod rain ellipse was shifted towards lower Δ^13^C and Δ^15^N values. Therefore, mean Δ^13^C and Δ^15^N values of soil invertebrates (4.0 ± 0.1‰ and 4.2 ± 0.1‰, respectively) were significantly higher than those of the arthropod rain (2.4 ± 0.1‰ and 1.5 ± 0.1‰, respectively) (Mann–Whitney test, P < 0.05). Due to the contribution of large specimens of spiders and especially harvestmen, weighted mean Δ^13^C and Δ^15^N values (according to the Eq. (3)) of the total arthropod rain were somewhat higher (2.7‰ and 2.4‰, respectively) (Table 1).

**Table 1:** Isotopic composition (litter-normalized Δ^13^C and Δ^15^N values) of soil animals (Potapov et al.^16^) and of arthropod rain and its main components (mean and weighted mean values).

	N	Δ^13^C, ‰	Δ^15^N, ‰
Soil animals^a^ (mean)	1300	4.0 (0.1)	4.2 (0.1)
**Arthropod rain** (mean)	**730**	**2.4 (0.1)**	**1.5 (0.1)**
Predators and parasitoids	206	3.1 (0.1)	3.6 (0.2)
Phytophages, saprophages and microphytophages	435	2.1 (0.1)	0.3 (0.2)
Winged animals	268	3.6 (0.1)	4.0 (0.2)
Wingless animals	429	1.8 (0.1)	0.1 (0.1)
**Arthropod rain** (weighted mean^b^)	**730**	**2.7 (0.1)**	**2.4 (0.1)**
Predators and parasitoids	206	2.7 (0.1)	3.2 (0.2)
Phytophages, saprophages and microphytophages	435	2.9 (0.1)	1.7 (0.2)
Winged animals	268	4.2 (0.1)	4.5 (0.2)
Wingless animals	429	1.8 (0.1)	1.3 (0.1)

Standard errors are shown in brackets.

^a^Potapov et al.^16^.

^b^According to the Eq. (3).

**Figure 2:**
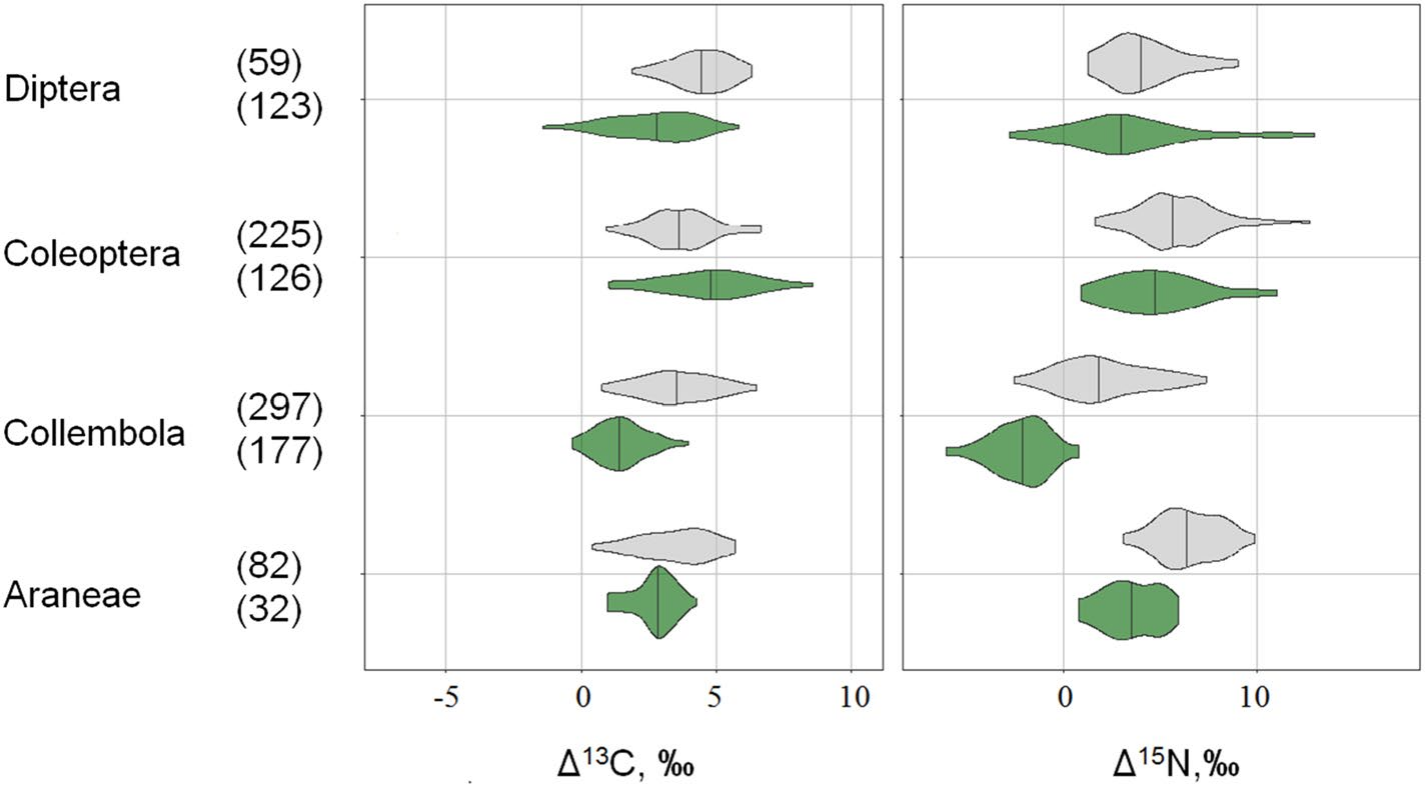
Stable isotope composition of arthropods from soil and litter (Potapov et al.^16^, gray) and the arthropod rain (original data, green). Litter-normalized Δ^13^C and Δ^15^N are shown as violin plots (mirrored kernel density estimation) with medians and 2.5–97.5% percentiles. Only groups represented by more than 30 data records are shown, and the number of samples is given in brackets. The difference within pairs was significant in all cases (Mann–Whitney test, P < 0.05).

As a rule, individual taxonomic groups forming arthropod rain had Δ^13^C and Δ^15^N values lower than those of animals collected from soil and litter. Significant differences both in Δ^13^C and Δ^15^N values between specimens originating from the arthropod rain (original data) and the soil (published data) were found in Diptera, Collembola, and Araneae (Fig. 2). Coleoptera from the arthropod rain were significantly depleted in ^15^N but enriched in ^13^C compared to soil-dwelling animals.

**Figure 3:**
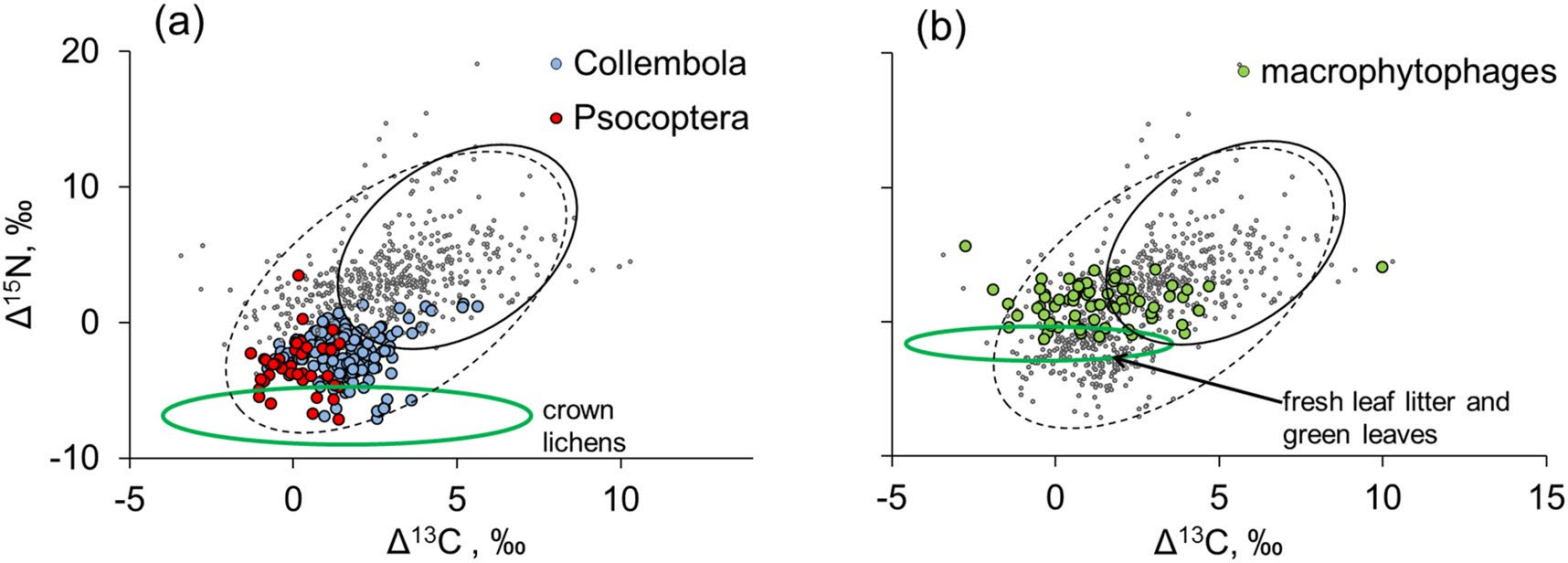
Isotopic composition (litter-normalized Δ^13^C and Δ^15^N values) of Collembola and Psocoptera (**a**) and macrophytophages (**b**) from the arthropod rain and of presumed food sources (lichens and green leaves and fresh leaf litter, green ellipses). Other animals forming arthropod rain are shown as gray dots. Each point is one specimen or several conspecifics. Standard ellipses for the arthropod rain and soil-dwelling invertebrates are shown as dashed and solid black lines, respectively.

Representatives of the arthropod rain that were most depleted in ^13^C and ^15^N comprised mainly microphytophages feeding on non-vascular plants and macrophytophages feeding on green leaves (Fig. 3). Microphytophages with low δ^13^C and δ^15^N values were represented primarily by Collembola (mean Δ^13^C and Δ^15^N values 1.5 ± 0.1‰ and − 2.2 ± 0.1‰, respectively, n = 177), and Psocoptera (mean Δ^13^C = 0.2 ± 0.1‰; mean Δ^15^N = − 3.2 ± 0.3‰, n = 40) (Fig. 3a). Macrophytophages, i.e. caterpillars, aphids, herbivorous heteropterans (Acanthosomatidae, Ligaeidae, Miridae, Tingidae), larvae and imagoes of beetles (Apionidae, Chrysomelidae, Curculionidae) and flies (Limoniidae, Agromyzidae) were depleted in ^13^C but not in ^15^N (mean Δ^13^C = 1.3 ± 0.2‰; Δ^15^N = 1.4 ± 0.2‰, n = 70) (Fig. 3b).

**Figure 4:**
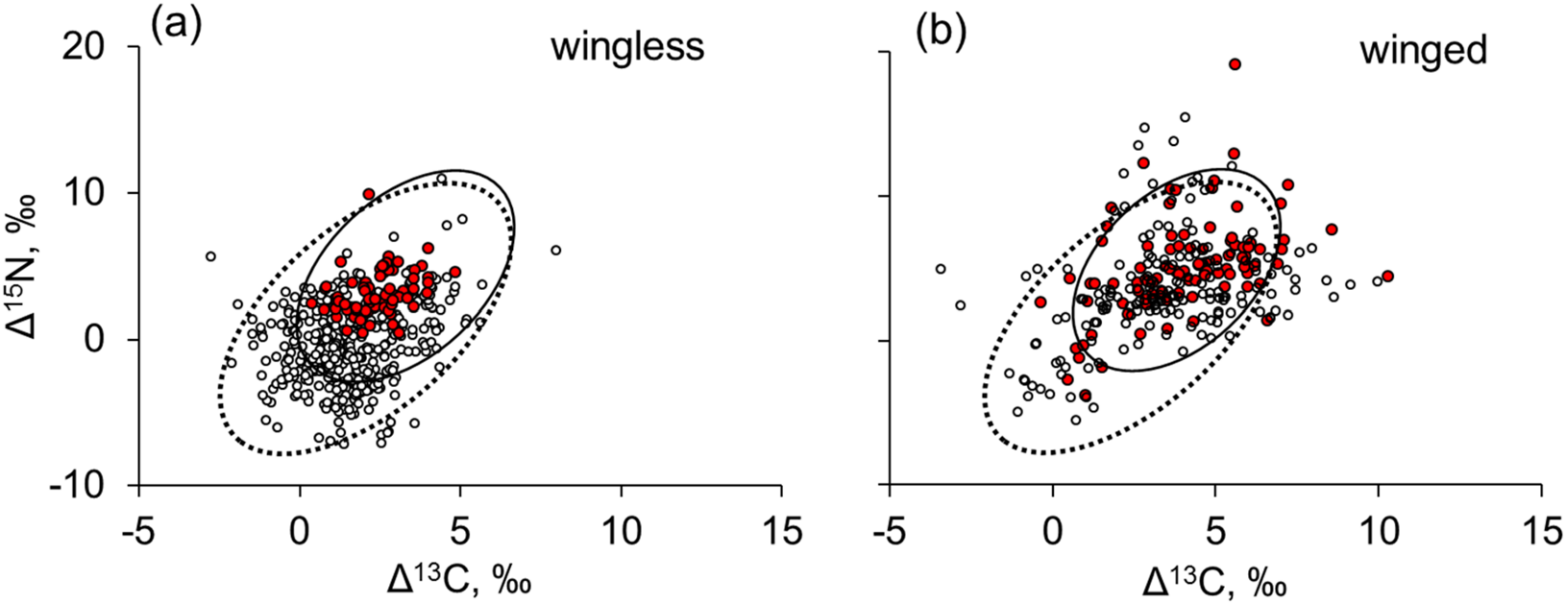
Stable isotope composition (litter-normalized Δ^13^C and Δ^15^N values) of wingless (**a**) and winged (**b**) arthropods within the arthropod rain throughout the vegetation season. Predators and parasitoids are shown as red dots, phytophages and microbi/saprophages as open dots. Standard ellipses for the arthropod rain and soil invertebrates are shown as dashed and solid black lines, respectively.

Wingless and winged invertebrates within the arthropod rain differed considerably in isotopic composition. Wingless arthropods were on average depleted in heavy isotopes relative to winged specimens (Table 1). The differences in both Δ^13^C and Δ^15^N values were significant. Among wingless invertebrates, predators and parasitoids differed significantly from phytophages and microbi/saprophages (Fig. 4a). In contrast, there was no difference in Δ values between saprophages and predators in winged insects. Winged insects were on average similar in the isotopic composition to soil-dwelling invertebrates (Fig. 4b, Table 1).

## Discussion

Arthropod rain sampled in two biotopes in a temperate mixed forest throughout vegetation season reflected a great taxonomic and functional diversity of crown fauna and air plankton. Seasonal changes in the abundance and taxonomic composition of the arthropod rain were reported elsewhere^12^. The stable isotope composition of the arthropod rain (730 samples) was compared to a large reference dataset of the isotopic composition of soil animals from temperate forests compiled in Potapov et al.^16^ (1300 samples). Both datasets contained litter-normalized Δ^13^C and Δ^15^N values, allowing a direct comparison of data collected in different biotopes^28^. As a note of caution, it should be stressed that sizes of the standard ellipses reflecting “isotopic space” of soil animals and arthropod rain (Fig. 1) cannot be compared directly, as they were based on species means and individual measurements, respectively. Nevertheless, their centroids can be accurately compared. This accuracy is confirmed by a close similarity in the isotopic signatures of soil macro- and mesofauna collected in this study and those represented in the reference dataset (Fig. S1).

Consistent with our first hypothesis, invertebrates forming arthropod rain were on average depleted in ^13^C and ^15^N compared to the soil-dwelling animals. Preservation of the arthropod rain invertebrates in 75% alcohol could not affect this conclusion since the expected change in ^13^C content due to leaching of lipids^29^ would increase, rather than decrease, the δ^13^C value of the arthropod rain.

The overall depletion of the arthropod rain in ^13^C was mainly driven by the presence of a significant proportion of macro- and microphytophages with relatively low δ^13^C values^15,30^. Furthermore, there was a clear difference between microphytophages and macrophytophages in δ^15^N values (Fig. 3), consistent with the difference in isotopic signatures of their basic trophic resources: non-vascular plants, such as algae and lichens, and fresh leaves, respectively. Indeed, the difference in Δ^15^N values between micro- and macrophytophages roughly corresponded to the difference between crown lichens and fresh leaf litter (Table S1). These data corroborate previous reports on the importance of non-vascular plants in forest food webs^20^.

Microphytophages depleted in ^15^N were represented mainly by Psocoptera and Collembola (Table S1). Psocoptera grazing on epiphytes are typical components of crown fauna^31^, while Collembola are usually regarded as typical soil animals feeding predominantly on fungi. Nevertheless, feeding of Collembola on ^15^N-depleted lower plants has been repeatedly noted. According to Potapov et al.^24^, at least 20% of Collembola species in temperate forest soils are depleted in ^15^N relative to litter, suggesting they are trophically linked to non-vascular plants, predominantly algae^20,32^. Thus, even in the soil, there are many phycophagous Collembola, but in the crowns, microphytophagy is apparently more widespread, as suggested by significantly lower δ^15^N and δ^13^C values in the Collembola from the arthropod rain than in soil-dwelling Collembola (Fig. 2).

Among other groups of arthropods well represented in both datasets, Diptera and Araneae were depleted in ^13^C compared to soil-dwelling animals. This observation further confirms that the “detrital shift,” i.e., enrichment of detrital food webs with ^13^C due to interactions with saprotrophic microorganisms (see Potapov et al.^16^ and references therein) can be traced both in micro- and macroarthropods and also at higher trophic levels. Coleopterans did not follow this pattern (Fig. 2) likely because they were represented mainly by winged imagoes trophically linked to detrital food webs (Table S1).

Dead stems and branches, bark crevices, suspended litter and soil support a substantial amount of detritus in the crown space, which in turn harbors rich fauna of detritophagous arthropods^33,34^. Thus, the detrital shift can be expected and was observed in the canopy food webs^35^. Nevertheless, the isotopic signature of non-winged specimens, which presumably fed in the crowns, suggests that the effect of the detrital shift in crown fauna was considerably less pronounced than in the soil food webs (Fig. 4a). Furthermore, soil-dwelling taxa associated with mineral soil that are the most enriched in ^13^C and ^15^N, such as earthworms and euedaphic Collembola among saprophages, or gamasid mites and geophilid centipedes among predators^16,36^, were rare or absent in our samples of the arthropod rain.

On the other hand, a large range of δ^13^C values in macrophytophages (ca. 8‰, Fig. 3b) can be related to the “canopy effect,” i.e., a gradient in the concentration of ^13^C in green leaves growing at different heights^37^. Therefore, phytophages that consumed green parts of vascular plants at different canopy heights could differ greatly in isotopic carbon composition.

As suggested by our second hypothesis, decreased δ^13^C and δ^15^N values were typical of wingless arthropods, while winged insects collected in the traps hardly differed in the isotopic composition from soil animals (Table 1, Fig. 4b). Another important feature of winged insects was the lack of difference between predators and phytophages or microbi/saprophages, while in the wingless arthropods, this difference was pronounced (Fig. 4). This observation confirms that winged insects collected in the traps represented a random assemblage of specimens originating in different biotopes or local ecosystems. Nevertheless, isotopic signatures of the winged insects suggest that they mostly originated from the soil. This localization is especially true for Diptera and Coleoptera (Table S1) that often have litter-dwelling larvae^25^. Thus, exploring the descending gravity-driven flow^11^ of arthropod rain, we found evidence of the ascending flow of the nutrients and energy from the soil to the crown layer.

The flux of arthropods falling from the crown space in temperate forests can be quite large. According to our calculations, its intensity is approximately 20 mg dry weight m^−2^ day^−1^ and can be comparable to the total food requirement of soil-dwelling spiders^12^. A significant proportion of the arthropod rain biomass (up to 40% in certain months) consists of small and slow-moving arthropods (such as Psocoptera, Aphidoidea, and Collembola), which can be easy prey for predators. Furthermore, approximately a third of arthropod rains consist of dead animals or their fragments that decomposers can consume. One of the objectives of this study was to assess the possibility of evaluating the contribution of arthropod rain to the nutrition of soil invertebrates using stable isotope analysis. The biomass-weighted mean values of Δ^13^C and Δ^15^N of the arthropod rain were approximately 1.3 and 1.8‰ lower, respectively, than the mean Δ^13^C and Δ^15^N values of soil animals. Even smaller differences have been used to identify energy pathways in detrital food webs^38,39^. However, soil food webs contain numerous mycrophytophages, e.g. Collembola, strongly depleted in ^13^C and ^15^N^20,24^, while the difference in the isotopic composition between soil animals and arthropod rain is likely not consistent in different forest types. In particular, it was less pronounced in a monsoon tropical forest (Rozanova et al., unpublished data). Thus the possibility of using isotopic composition of the arthropod rain to quantify its dietary inputs into soil food web remains questionable.

Overall, our data suggest that invertebrates falling from the crown space and flying arthropods originating from the soil are an important channel connecting food webs in the crown and the soil. Due to the large contribution of micro- and macrophytophages, the fraction of the arthropod rain consisting of wingless specimens differs considerably in δ^13^C and δ^15^N values from soil invertebrates belonging to detrital food webs.

## Methods

### Study site and sampling

The study was conducted in two forested plots near Malinky Biological Station (Moscow region, Russia, 55°27′42″ N, 37°11′10″ E) as described in Rozanova et al.^12^. The first plot was located in a mixed forest with spruce (*Picea abies* L.) and lime (*Tilia cordata* Mill.) forming the upper canopy. The second plot was situated nearby in a ca. 50-year-old pure *P. abies* plantation.

The arthropod sampling was conducted using six custom-made traps in each of the two plots. The traps were open for 24 h once every two weeks (± 3 days) throughout the growing season from May to October 2017 (12 samplings in total). Further details are given in Rozanova et al.^12^. Collected arthropods were preserved in 75% ethanol and subsequently identified to the order or family level. In addition to arthropods, fresh leaf litter and pollen were collected from the traps. Other sampled substrates included soil (upper 5 cm) and mixed leaf litter from the soil surface. Lichens, bark, tree branches, green leaves, and needles were sampled at different heights of the canopy trees (from 1 to 20 m) in July 2017. Isotopic composition and the number of replications for these substrates are given in Table S1. Five samples of soil and litter (25 × 25 cm, 10 cm deep) were taken in each study plot, and soil macrofauna was extracted by hand-sorting. These soil-dwelling animals were subsequently used for validating the reference dataset (see below).

The current study was conducted in accordance with guidelines of collecting biological materials for scientific purposes (Federal Law #200 of 04/12/2006). The research did not involve rare or endangered species of plants or animals. The collection of plant material complied with relevant institutional, national, and international guidelines and legislation. The appropriate permissions for collection of plant specimens were obtained for the study.

### Stable isotope analysis

All materials were dried at 50 °C for at least 72 h. Identified animals were weighed (dry wt.) individually or in batches of several conspecifics using a Mettler Toledo MX5 microbalance with 2 μg accuracy. For the isotope analysis of macrofauna, legs and/or head capsules of large arthropods were used^40^. Small animals were analyzed alone or as a group of several individuals from the same taxonomic group (minimum sample weight was approximately 50 μg). Soil and plant materials were ground to powder using an MM200 ball mill (Retsch, Germany). Stable isotope analyses were performed using a Flash 1112 Elemental Analyzer (Thermo Fisher, USA) and a Thermo Delta V Plus isotope ratio mass spectrometer in the Joint Usage Center “Instrumental Methods in Ecology” at the A.N. Severtsov Institute of Ecology and Evolution, Russian Academy of Sciences, Moscow. The carbon and nitrogen isotopic compositions were measured as deviations from the international standards (Vienna Pee Dee belemnite and atmospheric N_2_, respectively) and expressed in conventional δ values (‰):

1 $$\updelta {\text{X}}\, = \,\left[ {\left( {{\text{R}}_{{{\text{sample}}}} /{\text{R}}_{{{\text{standard}}}} } \right) \, {-} \, 1} \right] \, * \, 1000,$$

where X is the element of interest (carbon or nitrogen), and R is the molar ratio of its heavy and light isotopes. The standard deviations of δ^15^N and δ^13^C values in laboratory standards were < 0.15‰.

Preservation of sampled animals in 75% ethanol could affect their isotopic composition. In particular, the δ^13^C values could slightly increase due to the washing out of ^13^C-depleted lipids. However, the effect of ethanol preservation is typically small and does not exceed 1‰^41,42^. We, therefore, did not apply any correction related to the preservation of samples.

### Data analysis

The stable isotope composition of arthropods and litter collected at two experimental plots did not differ. Furthermore, we did not detect significant changes in the isotopic composition of animals during the growing season (data not shown). All materials collected were therefore analyzed together. Local δ^13^C and δ^15^N values of plant litter are typically used as a baseline in isotopic studies of soil-dwelling invertebrates^16,28^. To compare our results with published data, the isotopic composition of nitrogen and carbon of arthropods was therefore normalized using δ^13^C and δ^15^N values of fresh plant litter collected in the traps (δ^13^C − 28.9 ± 0.1‰, δ^15^N − 0.4 ± 0.1‰, n = 35; Table S1):

2 $$\Delta {\text{X}}_{{{\text{normalized}}}} = \updelta {\text{X}}_{{{\text{arthropod}}}} - \updelta {\text{X}}_{{{\text{litter}}}}$$

Dry-mass weighted mean δ^13^C and δ^15^N values of the arthropod rain were calculated using the following equation:

3 $${\text{Weighted mean}}\;\;\delta {\text{X}} = \sum\limits_{i = 1}^{n} {w_{i} \cdot \;\delta X_{i} } ,$$

where $w_{i} = \frac{{m_{i} }}{{\sum\nolimits_{i = 1}^{n} {m_{i} } }}$, i.e., mass proportion of the individual samples in a total flux, *m*_*i*_—dry mass of *i*-th of *n* samples from a group of the arthropod rain and δX_*i*_—isotope signatures (δ^13^C or δ^15^N) of the individual sample.

The trophic position of individual taxonomic groups of arthropods was derived based on the known morphological and behavioral traits^43^ and isotopic studies^16^. Some groups of arthropods that have fallen into our traps can move between the forest canopy and the litter layer. These are winged animals (the imago stage of various insects, marked in Table S1) and actively moving wingless predators, such as ants, harvestmen, and spiders. We, therefore, separated winged animals and predators from other arthropods.

We compared the isotopic composition of the arthropod rain (original data, n = 730) to that of soil invertebrates using the results of 23 studies performed in temperate forests compiled in Potapov et al.^16^ (published results, n = 1300). Although the dataset used for comparison^16^ contains averaged values rather than individual measurements, it fully reflects data on the isotopic composition of soil and litter-dwelling mesofauna and macrofauna in the vicinity of the Malinky Biological Station (Fig. S1). In addition, we compared the isotopic composition of high-rank taxonomic groups that were well represented (more than 30 measurements) in the original and published datasets (Diptera, Coleoptera, Collembola, and Araneae).

The isotopic composition of arthropod rain and soil-dwelling invertebrates was compared using standard ellipses limiting the 95% confidence interval^44^. The area and the overlap of the ellipses were calculated in the SIBER package, and violin plots (mirrored kernel density estimation) were produced in the ggplot2 package in R^45^. Central tendencies are presented as means ± 1 SE. Pairwise comparisons were performed in STATISTICA 10 (StatSoft, Tulsa, USA) using Mann–Whitney U test. P < 0.05 was considered statistically significant.

## Supplementary Information

Supplementary Information.

## Data Availability

Should the study be accepted for publication, the original dataset will be placed in an open repository (Figshare.com or similar).
