# Supplementary material for: The isotopic signature of the “arthropod rain” in a temperate forest
Source: Sci Rep. 2022 Jan 10;12:321. doi: 10.1038/s41598-021-03893-6 (PMC8748442; doi:10.1038/s41598-021-03893-6)
Supplement: Supplementary file 1 — Supplementary Information. [file 41598_2021_3893_MOESM1_ESM.docx]

**Supplementary materials**


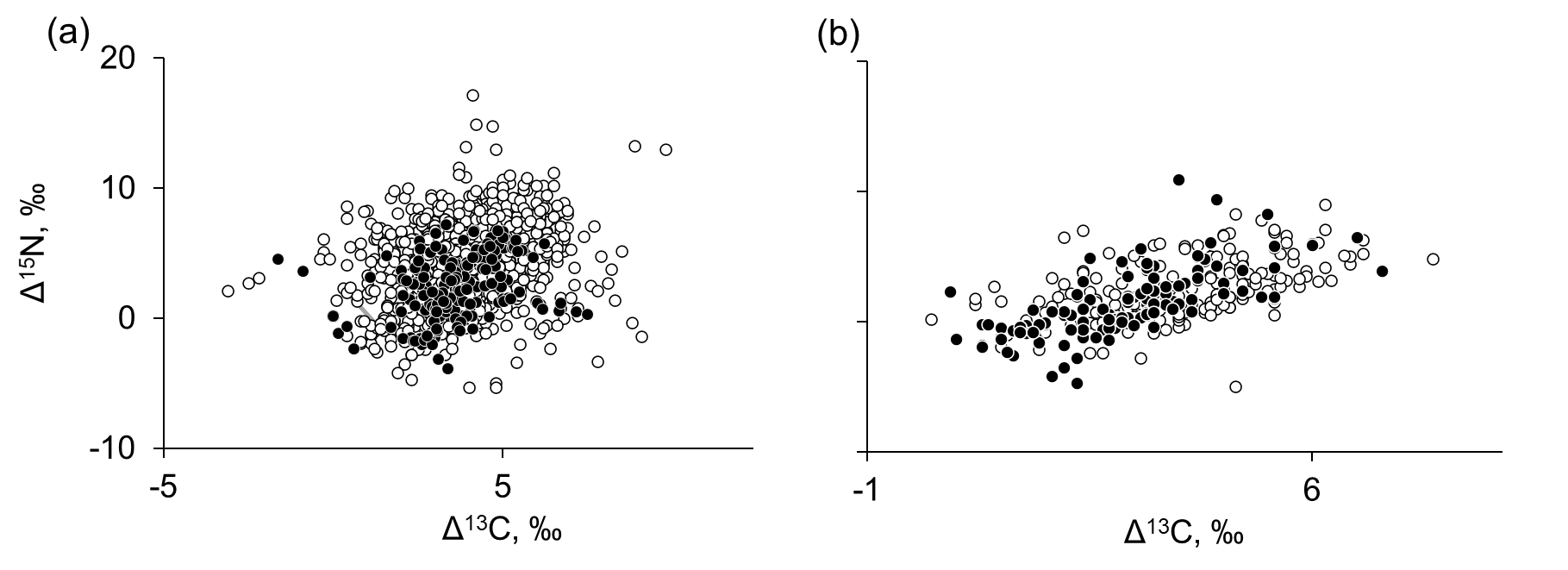


Fig. S1. (a) Isotopic composition (litter-normalized δ^13^С and δ^15^N values) of soil and litter-dwelling animals collected at Malinky Biological Station during this study (black dots, each point is one animal, n = 165) and those reported in Potapov et al. (2019) (open dots, points represent mean value of species in different ecosystems, n = 1300). (b) Isotopic composition of soil-dwelling Collembola. All data are taken from Potapov et al. (2019). Closed dots: Collembola collected at Malinky Biological Station (n = 114). Open dots: Collembola collected in other temperate forests (n = 183).


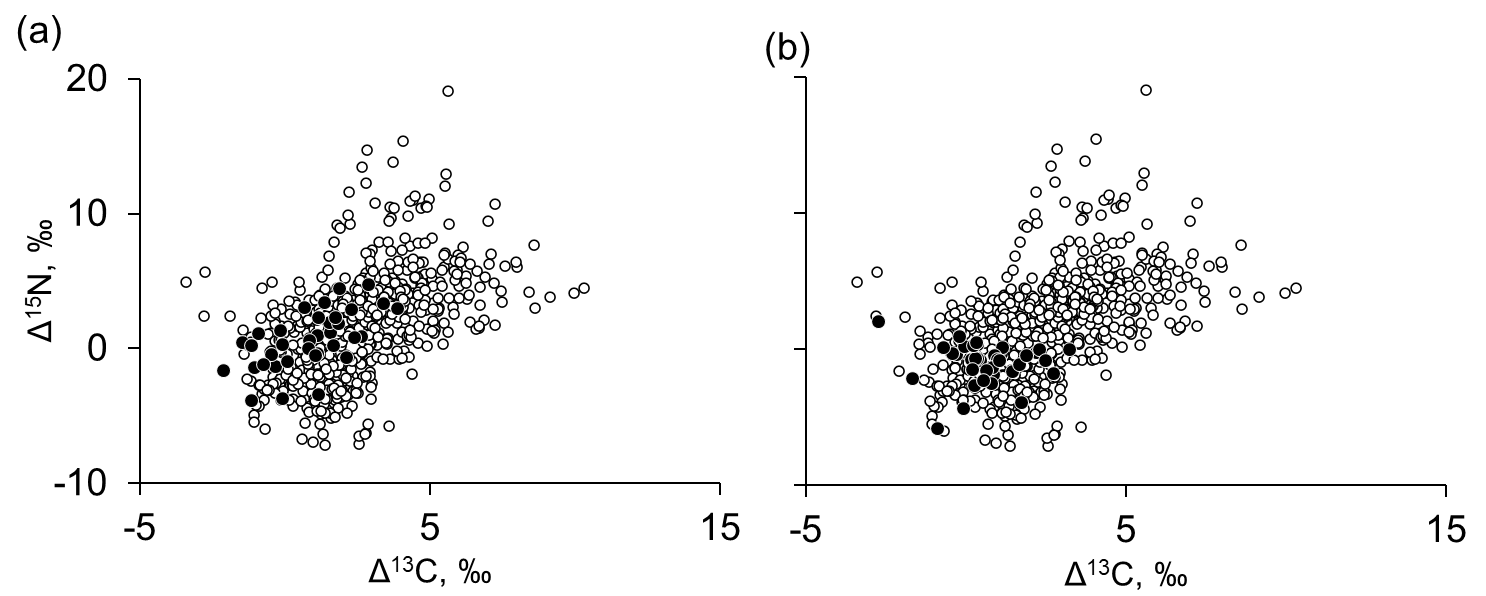


Fig. S2. Isotopic composition (litter-normalized δ^13^C and δ^15^N values) of exuviae ((a), n = 35), and excrements ((b), n = 36) collected in the traps (closed dots). Isotopic composition of arthropods from the arthropod rain is shown as open dots.

Table S1. Stable isotope composition (not normalized δ^13^C and δ^15^N values) of soil and plant materials, and of individual taxa of animals forming the arthropod rain in two forest plots. Unweighted mean values ± 1 SE, data are averaged across two forest plots and a whole growing season.

| Material / Taxa / Order | | | | Family / Stage | | Spruce forest | | | Mixed forest | | |
| --- | --- | --- | --- | --- | --- | --- | --- | --- | --- | --- | --- |
|  |  |  |  |  |  | δ^13^C, ‰ | δ^15^N, ‰ | n | δ^13^C, ‰ | δ^15^N, ‰ | n |
| Fresh leaf litter from the traps | | | |  |  | -29.0±0.1 | -0.1±0.1 | 18 | -28.8±0.2 | -0.7±0.1 | 17 |
| Leaf litter from the soil surface | | | |  |  | -29.8±0.3 | 0.3±0.4 | 3 | -28.6±0.1 | -0.8±0.3 | 3 |
| Green needles | | | |  |  | -30.6±1.2 | 0.5±0.7 | 6 | -32.5±0.5 | -0.3±0.4 | 3 |
| Green leaves | | | |  |  | na | | | -31.7±0.5 | -0.9±0.8 | 5 |
| Bark | | | |  |  | -28.6±0.3 | -0.3±0.4 | 18 | -28.5±0.3 | -2.1±0.6 | 10 |
| Wood | | | |  |  | -30.6±0.4 | -0.7±0.2 | 3 | -31.6±0.7 | -1.2±0.2 | 3 |
| Pollen | | | |  |  | -25.7±0.1 | 0.6±0.1 | 3 | -25.3±0.0 | -0.8±0.1 | 4 |
| Lichens | | | |  |  | -30.4±1.0 | -4.0±0.4 | 14 | -29.8±0.6 | -6.8±0.8 | 8 |
| Soil | | | |  |  | -28.6±0.2 | 1.4±0.7 | 6 | -27.3±0.2 | 1.6±1.6 | 5 |
| Excrements (frass) | | | |  |  | -28.5±0.3 | -0.7±0.3 | 18 | -28.0±0.3 | -2.1±0.3 | 18 |
| **Total Arthropod rain** | | | **mean** |  |  | **-27.0±0.1** | **1.1±0.2** | **349** | **-26.0±0.1** | **1.0±0.2** | **351** |
|  | **exuviae** | | **mean** |  |  | **-28.4±0.3** | **0.3±0.5** | **19** | **-27.6±0.4** | **0.2±0.5** | **16** |
|  | **Acari** | | **mean*** |  |  | **combined** | | | **-26.7±0.5** | **2.1±0.6** | **16** |
|  |  | Astigmatina* |  |  |  | combined | | | -26.5±0.5 | 4.4±0.0 | 3 |
|  |  | Gamasina* |  |  |  | combined | | | -24.8±2.0 | 3.7±1.0 | 3 |
|  |  | Oribatida* |  |  |  | combined | | | -27.6±0.5 | -0.7±1.1 | 3 |
|  |  | Trombidiformes* |  |  |  | combined | | | -27.3±0.5 | 3.0±0.8 | 5 |
|  | **Araneae** | | **mean** |  |  | **-26.7±0.3** | **3.6±0.5** | **13** | **-25.8±0.2** | **2.6±0.3** | **19** |
|  |  |  |  |  | adult (total) | -26.6±0.5 | 4.7±0.2 | 3 | -25.9±0.2 | 2.4±0.6 | 9 |
|  |  |  |  |  | juvenile (total) | -26.7±0.3 | 3.3±0.6 | 10 | -25.7±0.3 | 3.1±0.4 | 8 |
|  |  |  |  | Araneidae | juvenile | -26.5±0.6 | 3.6±1.0 | 4 | -25.0±0.6 | 4.4±0.1 | 3 |
|  |  |  |  | Linyphiidae | total | -26.6±0.5 | 5.0±0.1 | 3 | -26.3±0.2 | 2.5±0.3 | 4 |
|  |  |  |  | - | adult | -26.9±0.7 | 4.8±0.0 | 2 | -26.1±0.1 | 2.8±0.1 | 3 |
|  |  |  |  | - | juvenile | -26.1 | 5.2 | 1 | -26.8 | 1.5 | 1 |
|  |  |  |  | Philodromidae | adult | na | | | -25.3 | 1.8 | 1 |
|  |  |  |  | Salticidae | adult | -26.1 | 4.4 | 1 | na | | |
|  |  |  |  | Tetragnathidae | adult | na | | | -25.3 | 4.3 | 1 |
|  |  |  |  | Theridiidae | total | -27.2±0.6 | 2.2±0.7 | 4 | -26.3±0.3 | 1.1±0.4 | 5 |
|  |  |  |  | - | adult | na | | | -26.3±0.5 | 0.4±0.3 | 3 |
|  |  |  |  | - | juvenile | -27.2±0.6 | 2.2±0.7 | 4 | -26.4±0.2 | 2.0±0.2 | 2 |
|  |  |  |  | Thomisidae | total | -26.4 | 4.4 | 1 | -25.6±0.1 | 3.5±0.5 | 4 |
|  |  |  |  | - | adult | na | | | -25.6±0.2 | 4.0±0.9 | 2 |
|  |  |  |  | - | juvenile | -26.4 | 4.4 | 1 | -25.6±0.1 | 2.9±0.0 | 2 |
|  | **Coleoptera** | | **mean** |  |  | **-24.2±0.2** | **4.9±0.3** | **55** | **-24.1±0.2** | **4.2±0.3** | **69** |
|  |  |  |  | 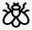 | imago (total) | -24.1±0.3 | 4.7±0.4 | 50 | -23.9±0.2 | 4.4±0.3 | 62 |
|  |  |  |  |  | larva (total) | -25.4±0.8 | 6.3±1 | 4 | -25.8±0.6 | 1.9±0.6 | 7 |
|  |  |  |  | Apionidae 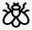 | imago | na | | | -27.3 | 1.7 | 1 |
|  |  |  |  | Cerylonidae 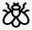 | imago | -23.2±0.5 | 4.8±0.1 | 3 | na | | |
|  |  |  |  | Cholevidae 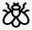 | imago | -23.9 | 10.7 | 1 | -23.3 | 12.5 | 1 |
|  |  |  |  | Chrysomelidae 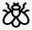 | imago | -28.0±0.5 | 1.1±0.2 | 2 | na | | |
|  |  |  |  | Ciidae 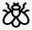 | imago | na | | | -22.5 | 1.6 | 1 |
|  |  |  |  | Coccinellidae 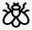 | imago | na | | | -28.4 | 3.9 | 1 |
|  |  |  |  | Corylophidae 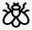 | imago | -23.6±0.8 | 5.2±0.6 | 6 | -21.7 | 4.5 | 1 |
|  |  |  |  | Cryptophagidae 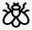 | imago | -23.6±0.2 | 2.3±2.4 | 2 | -22.5±1.1 | 3.2±0.6 | 2 |
|  |  |  |  | Curculionidae 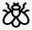 | imago | -25.8±0.7 | 1.4±0.0 | 2 | -25.9 | 3.5 | 1 |
|  |  |  |  | Latridiidae 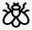 | imago | -23.9±0.5 | 6.7±1.8 | 4 | -23.6±0.4 | 5.8±0.2 | 6 |
|  |  |  |  | Leiodidae 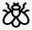 | imago | -21.7±1.1 | 2.5±0.6 | 3 | -22.2 | 1.1 | 1 |
|  |  |  |  | Monotomidae 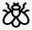 | imago | na | | | -23.7±0.3 | 2.0±0.5 | 2 |
|  |  |  |  | Peltidae 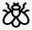 | imago | -23.8 | 1.5 | 1 | -20.3 | 2.6 | 1 |
|  |  |  |  | Pselaphidae 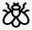 | imago | -24.2±0.7 | 4.9±0.5 | 2 | -22.0 | 4.9 | 1 |
|  |  |  |  | Ptiliidae 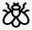 | imago | -24.3 | 0.0 | 1 | -23.7±1.2 | 3.1±1.2 | 3 |
|  |  |  |  | Pythidae 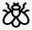 | imago | na | | | -25.2±0.6 | 5.9±3.5 | 2 |
|  |  |  |  | Scydmaenidae 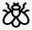 | imago | -24.6 | 7.2 | 1 | na | | |
|  |  |  |  | Silvanidae 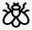 | imago | -23.3±1.9 | 4.0±0.9 | 2 | -23.3±1.2 | 3.8±1.1 | 3 |
|  |  |  |  | Staphylinidae 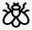 | imago | -24.4±0.4 | 5.4±0.4 | 20 | -24.1±0.4 | 4.9±0.4 | 31 |
|  |  |  |  | Zopheridae 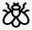 | imago | na | | | -25.3 | 3.0 | 1 |
|  | **Collembola** | | **mean** |  |  | **-27.8±0.1** | **-1.9±0.1** | **87** | **-26.9±0.1** | **-3.6±0.2** | **83** |
|  |  | Symphypleona |  | Dicyrtomidae |  | -27.9±0.1 | -3.9±1.1 | 4 | -26.6±0.2 | -3.2±0.3 | 16 |
|  |  |  |  | Sminthuridae |  | -28.1±0.1 | -2.3±0.3 | 17 | -26.7±0.2 | -4.9±0.4 | 15 |
|  |  | Entomobryomorpha |  | Entomobriydae |  | -28.1±0.2 | -1.8±0.1 | 38 | -27.1±0.1 | -3.5±0.2 | 47 |
|  |  |  |  | Isotomidae |  | na | | | -26.3 | -1.5 | 1 |
|  |  |  |  | Tomoceridae |  | na | | | -27.0 | -1.7 | 1 |
|  |  | Poduromorpha |  |  |  | -27.3±0.3 | -1.4±0.2 | 28 | -26.7±0.8 | -1.1±1.4 | 2 |
|  | **Diptera** | | **mean** |  |  | **-26.3±0.2** | **3.1±0.5** | **63** | **-26.3±0.3** | **2.7±0.6** | **48** |
|  |  |  |  | 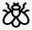 | imago (total) | -25.9±0.3 | 5.0±0.7 | 32 | -25.9±0.4 | 4.1±0.6 | 33 |
|  |  |  |  |  | larva (total) | -26.7±0.3 | 1.2±0.4 | 31 | -27.1±0.3 | -0.3±0.6 | 15 |
|  |  |  |  | Agromyzidae | larva | -24.5±0.3 | 2.1±0.1 | 2 | na | | |
|  |  |  |  | Anisopodidae | larva | na | | | -30.5±1.8 | 2.8±1.7 | 2 |
|  |  |  |  | Calliphoridae | larva | -26.7 | 2.9 | 1 | na | | |
|  |  |  |  | Cecidomyiidae | total | -26.7±0.4 | 1.0±0.5 | 26 | -27.3±0.3 | -0.4±0.6 | 16 |
|  |  |  |  | 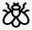 | imago | -24.9±0.5 | 2.5±1.0 | 5 | -27.5±0.5 | 0.3±1.2 | 4 |
|  |  |  |  |  | larva | -27.1±0.4 | 0.6±0.5 | 21 | -27.3±0.3 | -0.7±0.7 | 12 |
|  |  |  |  | Ceratopogonidae 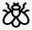 | imago | -26.7 | 8.8 | 1 | -27.6±0.3 | 1.3±0.7 | 2 |
|  |  |  |  | Chironomidae 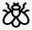 | imago | -26.3 | 1.6 | 1 | -26.5 | 3.2 | 1 |
|  |  |  |  | Chloropidae 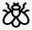 | imago | na | | | -24 | 10.2 | 1 |
|  |  |  |  | Drosophilidae 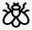 | imago | na | | | -25.7 | 1.7 | 1 |
|  |  |  |  | Empididae 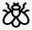 | imago | -27.9 | -0.7 | 1 | -24.7±0.6 | 6.0±1.4 | 2 |
|  |  |  |  | Limoniidae 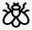 | imago | -27.1 | 3.1 | 1 | -23.8±2.5 | 2.6±0.6 | 3 |
|  |  |  |  | Lonchaeidae | total | -25.6±0.7 | 3.4±0.4 | 4 | -24.6±1.7 | 5.5±0.8 | 3 |
|  |  |  |  | 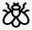 | imago | -26.7±0.7 | 3.1±0.9 | 2 | -24.6±1.7 | 5.5±0.8 | 3 |
|  |  |  |  |  | larva | -24.5±0.2 | 3.7±0.1 | 2 | na | | |
|  |  |  |  | Muscidae 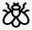 | imago | na | | | -26.7 | 2.2 | 1 |
|  |  |  |  | Mycetophilidae 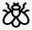 | imago | -25.1±0.3 | 11.1±2.1 | 3 | -25.8±0.5 | 7.4±5.7 | 2 |
|  |  |  |  | Psychodidae 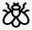 | imago | na | | | -27.0 | 8.6 | 1 |
|  |  |  |  | Scatopsidae 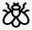 | imago | -28.6±3.1 | 3.6±1.6 | 2 | na | | |
|  |  |  |  | Sciaridae | total | -25.6±0.4 | 5.2±1.0 | 13 | -25.3±0.3 | 3.9±1.1 | 10 |
|  |  |  |  | 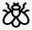 | imago | -25.6±0.4 | 5.2±1.0 | 13 | -25.4±0.3 | 4.0±1.2 | 9 |
|  |  |  |  |  | larva | na | | | na | | |
|  |  |  |  | Syrphidae | larva | -29.0 | 1.3 | 1 | na | | |
|  | **Hemiptera** | | **mean** |  |  | **-28.2±0.2** | **1.3±0.3** | **36** | **-26.4±0.3** | **0.9±0.3** | **48** |
|  |  | Heteroptera | mean |  |  | -26.1±1 | 3.1±2 | 3 | -25.2±0.4 | 1.7±0.4 | 21 |
|  |  |  |  | Acanthosomatidae 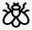 | adult | -25.4 | 0.0 | 1 | -26.5±0.2 | -0.8±0.2 | 2 |
|  |  |  |  | Aradidae | juvenile | na | | | -24.5±0.5 | 2.3±0.4 | 15 |
|  |  |  |  | Lygaeidae 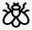 | adult | na | | | -26.7±0.8 | 0.8±0.1 | 2 |
|  |  |  |  | Miridae 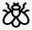 | adult | na | | | -27.1 | 2.8 | 1 |
|  |  |  |  | Reduviidae 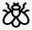 | adult | -24.8 | 6.9 | 1 | -27.4 | -2.3 | 1 |
|  |  |  |  | Tingidae 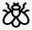 | adult | -27.9 | 2.5 | 1 | na | | |
|  |  | Others |  | Aphidoidea ^partly^ 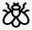 |  | -28.4±0.2 | 1.0±0.2 | 29 | -27.7±0.4 | 0.2±0.4 | 21 |
|  |  |  |  | Cicadellidae 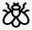 |  | -28.7±1.0 | 1.8±2.2 | 2 | na | | |
|  |  |  |  | Phylloxeridae 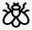 |  | na | | | -27.3 | 1.8 | 1 |
|  |  |  |  | Psyllidae 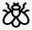 |  | -28.2±0.2 | 3.5±1.1 | 2 | -25.8±1.5 | 0.1±0.6 | 4 |
|  | **Hymenoptera** | | **mean** |  |  | **-24.7±0.6** | **6.1±2.2** | **7** | **-26.1±0.3** | **5.6±0.8** | **16** |
|  |  |  |  | Aphidiidae/Ephedrinae*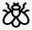 | imago | combined | | | -26.0 | 2.1 | 1 |
|  |  |  |  | Aphidiidae/Aphidiinae 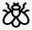 | imago | na | | | -24.6 | 1.0 | 1 |
|  |  |  |  | Braconidae 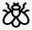 | imago | na | | | -25.3 | 9.1 | 1 |
|  |  |  |  | Ceraphronidae* 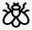 | imago | combined | | | -26.0 | 6.1 | 1 |
|  |  |  |  | Diapriidae 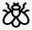 | imago | -22.5 | 3.6 | 1 | -24.8±0.4 | 10.1±0.0 | 3 |
|  |  |  |  | Encyrtidae 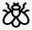 | imago | -26.0 | 2.1 | 1 | na | | |
|  |  |  |  | Figitidae 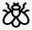 | imago | -23.3 | 18.7 | 1 | na | | |
|  |  |  |  | Formicidae | imago | na | | | -26.7±0.1 | 4.5±0.9 | 7 |
|  |  |  |  | Ichneumonidae 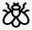 | imago | -26.3±0.7 | 3.7±0.2 | 2 | na | | |
|  |  |  |  | Megaspilidae 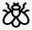 | imago | na | | | -25.3±0.0 | 6.4±0.5 | 2 |
|  |  |  |  | Mymaridae 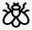 | imago | na | | | -26.3 | 3.6 | 1 |
|  |  |  |  | Platygastridae 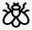 | imago | -25.1 | 4.4 | 1 | na | | |
|  |  |  |  | Pteromalidae 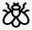 | imago | -23.4 | 6.5 | 1 | na | | |
|  | **Lepidoptera** | | **mean** |  | **larva (total)** | **-29.3** | **-1.7** | **1** | **-26.8±0.8** | **2.6±0.4** | **3** |
|  |  | |  | Argyresthidae | larva | na | | | -28.2 | 2.2 | 1 |
|  |  | |  | Geometridae | larva | na | | | -26.8 | 3.4 | 1 |
|  |  | |  | Lymantriidae | larva | -29.3 | -1.7 | 1 | na | | |
|  |  | |  | Tortricidae | larva | na | | | -25.4 | 2.3 | 1 |
|  | **Neuroptera** | | **mean** |  |  | **-29** | **0.2** | **1** | **-28.5±0.4** | **-2.3±1.3** | **3** |
|  |  |  |  |  | **imago (total)** | na | | | -28.2±0.3 | -3.6±0.5 | 2 |
|  |  |  |  | Chrysopidae | larva | -29.0 | 0.2 | 1 | -29.1 | 0.2 | 1 |
|  |  |  |  | Coniopterygidae 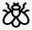 | imago | na | | | -28.4 | -3.1 | 1 |
|  | **Opiliones** | | **mean** |  | **adult** | **-27.4±0.1** | **1.7±0.2** | **15** | **-26.0±0.2** | **2.3±0.3** | **15** |
|  | **Plecoptera** | | **mean** |  | **larva** | **-29.7** | **1.9** | **1** | **na** | | |
|  | **Psocoptera** | | **mean** | ^partly^ 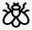 |  | **-28.9±0.1** | **-3.4±0.2** | **33** | **-27.8±0.1** | **-5.5±0.8** | **6** |
|  | **Thysanoptera** | | **mean** | ^partly^ 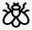 |  | **-25.4±0.4** | **2.8±0.2** | **9** | **-25.8±0.1** | **2.2±0.1** | **24** |

* Due to the low biomass, samples obtained in two forest plots were combined.


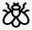
 Winged insects.
